# Maladaptive Synaptic Plasticity in L-DOPA-Induced Dyskinesia

**DOI:** 10.3389/fncir.2016.00105

**Published:** 2016-12-20

**Authors:** Qiang Wang, Wangming Zhang

**Affiliations:** The National Key Clinic Specialty, Guangdong Provincial Key Laboratory on Brain Function Repair and Regeneration, The Engineering Technology Research Center of Education Ministry of China, Department of Neurosurgery, Zhujiang Hospital, Southern Medical UniversityGuangzhou, China

**Keywords:** Parkinson disease, synaptic plasticity, dopamine, basal ganglia, motor cortex

## Abstract

The emergence of L-DOPA-induced dyskinesia (LID) in patients with Parkinson disease (PD) could be due to maladaptive plasticity of corticostriatal synapses in response to L-DOPA treatment. A series of recent studies has revealed that LID is associated with marked morphological plasticity of striatal dendritic spines, particularly cell type-specific structural plasticity of medium spiny neurons (MSNs) in the striatum. In addition, evidence demonstrating the occurrence of plastic adaptations, including aberrant morphological and functional features, in multiple components of cortico-basal ganglionic circuitry, such as primary motor cortex (M1) and basal ganglia (BG) output nuclei. These adaptations have been implicated in the pathophysiology of LID. Here, we briefly review recent studies that have addressed maladaptive plastic changes within the cortico-BG loop in dyskinetic animal models of PD and patients with PD.

## Introduction

Parkinson’s disease (PD) is characterized by severe, progressive degeneration of nigrostriatal dopamine (DA) neurons, which results in motor deficits, including akinesia, rigidity, tremor and postural dysfunction. These clinical manifestations can be ameliorated by pharmacological stimulation of DA biosynthesis with exogenous supplementation of L-DOPA (i.e., levodopa), the metabolic precursor to DA. Unfortunately, however, most patients who take L-DOPA also experience adverse secondary effects, including L-DOPA-induced dyskinesia (LID; Voon et al., 2009).

The striatum is the principal recipient of cortical efferents within the basal ganglia (BG). Hence, it serves as a main entryway of information from the neocortex to the BG in the BG-thalamo-cortical network. Furthermore, the striatum, which is composed of functionally and anatomically distinct dorsal and ventral divisions, is also the primary target of DA neurons of the substantia nigra pars compacta. In recent decades, impaired striatal function due to maladaptive synaptic plasticity has been implicated mechanistically in several movement disorders, including LID (Picconi et al., 2003; Paillé et al., 2010).

Recent methodological breakthroughs, such as bacterial artificial chromosome (BAC) and optogenetic techniques, have enabled researchers to not only mimic features of motor disorders in a controlled manner in relation to the extent of nigrostriatal degeneration, but also to further delineate and examine the functions of the direct- and indirect-pathway medium spiny neurons (MSNs; also known as spiny projection neurons) in the striatum (Cui et al., 2013; Fieblinger et al., 2014).

Apart from affecting corticostriatal synaptic plasticity, L-DOPA induced adaptions elsewhere in cortico-basal ganglionic circuitry have come to light (Cenci and Lundblad, 2006). Indeed, several studies have produced evidence suggesting that maladaptive synaptic plasticity processes throughout the cortico-basal ganglionic circuitry may be of critical importance to the pathophysiology of LID (Prescott et al., 2014; Ueno et al., 2014). The latest seminal studies addressing these plastic changes in dyskinetic animal models and patients are discussed below.

## DA-Dependent Synaptic Plasticity in Corticostriatal Synapses

The neuronal population in the striatum consists in large majority (~95%) of MSNs, which are projection cells characterized by the spiny cytoarchitecture of their dendritic trees, where synaptic plasticity occurs (Cenci et al., 2011). Striatal MSNs can be divided into two similarly sized populations based on their axonal projections: direct pathway SPNs (dMSNs) and indirect pathway MSNs (iMSNs).

Two forms of plasticity in corticostriatal synapses on MSNs have been characterized extensively long-term potentiation (LTP) and long-term depression (LTD). LTP and LTD describe persistent changes in the efficacy of synaptic transmission that are induced by repetitive activation of cortical excitatory afferents. A unique characteristic of striatal MSNs is that DA plays a critical role in both the induction and maintenance of their neuroplasticity.

Activity-dependent LTD induction is associated with the postsynaptic generation of endocannabinoids (eCBs). This eCB-dependent form of LTD (eCB-LTD) is seen in D2R-expressing iMSNs, but not D1R-expressing dMSNs (Kreitzer and Malenka, 2007). Meanwhile, activation of D2Rs on iMSNs restrains local type 2a adenosine receptor (A2AR) signaling. Spike-timing-dependent plasticity experiments have suggested that local A2AR activation can inhibit both eCB synthesis and LTD induction (Shen et al., 2008). Signaling via D2Rs and A2ARs appears to be linked to eCB-LTD through cAMP/protein kinase A (PKA) and regulator of G protein signaling 4 (RGS4; Lerner and Kreitzer, 2012).

LTP can be induced in MSNs by high-frequency stimulation (HFS) of glutamatergic inputs that results in co-activation of D1Rs and N-methyl-D-aspartate type glutamate receptors (NMDARs; Calabresi et al., 2007; Surmeier et al., 2014). In striatal MSNs, D1Rs co-localize with NMDARs and form heteromeric complexes on dendritic spines (Fiorentini et al., 2006; Calabresi et al., 2010). These D1R/NMDAR complexes facilitate rapid trafficking of NMDAR subunits and modulate the potentiation of NMDAR responses, giving rise to activity-dependent synaptic plasticity changes that involve PKA and dopamine- and cAMP-regulated phosphoprotein (DARPP)-32-regulated phosphorylation of the NR1 subunits of NMDARs (Fiorentini et al., 2008; Murphy et al., 2014).

In experimental models of the LID, a form of synaptic plasticity known as depotentiation has been observed; depotentiation reverses LTP and may represent a homeostatic mechanism (Picconi et al., 2003, 2008). Although the specific mechanisms responsible for homeostatic depotentiation are not yet known, several recent studies have provided important pieces of information (Table 1). One of the most reproducible observations has been that positive allostericmodulator, which modulates M4Rs, enables depotentiation in dSPNs by suppressing RGS4 signaling (Shen et al., 2015). Additionally, extracellular signal-regulated kinase (ERK) signaling has been reported to facilitate depotentiation under normal (unaltered) conditions, but to oppose it under dyskinetic conditions (Cerovic et al., 2015). Striatal synaptic depotentiation could be restored in a subset of striatal MSNs by 5-HT1A/1B receptor agonism with eltoprazine via a mechanism that involved normalization of D1R-dependent cAMP/PKA and ERK/mTORC signaling pathways and recovery of NMDAR subunit balance (Ghiglieri et al., 2016). Mice lacking D-aspartate oxidase (Ddo^−/−^) display high levels of free D-aspartate and NMDA, which stimulate NMDAR transmission; a low-frequency stimulation protocol failed to depotentiate HFS-induced LTP in Ddo^−/−^ mice (Errico et al., 2011). Finally, nociceptin/orphanin FQ, the endogenous agonist of the nociception receptor, has been shown to prevent D1R agonism-induced ERK phosphorylation and loss of depotentiation in MSNs (Marti et al., 2012). These findings provide insights into the mechanism of striatal neuron depotentiation and could, eventually, lead to novel therapeutic strategies for alleviating LID.

**Table 1:** **Summary of maladaptive depotentiation plasticity in cortico-basal ganglionic circuitry in LID**.

Brain area	Reference*	Synaptic mechanisms	Methods	Experimental conditions
d-Str	Ghiglieri et al. (2016)	cAMP/PKA, ERK/mTORC signaling pathways, NMDAR subunit imbalance	*Ex vivo*, intracellular recordings with electrodes, HFS, LFS	Unilateral 6-OHDA-induced lesion, LID rats
	Shen et al. (2015)	Abnormal M_4_R signaling pathway and RGS4 activity in dMSNs	*Ex vivo*, intracellular recordings with electrodes, HFS, LFS	Unilateral 6-OHDA-induced lesion, LID mice
	Cerovic et al. (2015)	Hyperactivation of Ras-ERK signaling pathway in dMSNs	*Ex vivo*, intracellular recordings with electrodes, HFS, LFS	Unilateral 6-OHDA-induced lesion, LID mice
	Marti et al. (2012)	N/OFQ and increased ERK phosphorylation	*Ex vivo*, intracellular recordings with electrodes, HFS, LFS	D1R agonist-induced LID rats
	Errico et al. (2011)	Abnormal high levels of D-Asp and NMDA	*Ex vivo*, intracellular recordings with electrodes, HFS, LFS	Unilateral 6-OHDA-induced lesion, *Ddo^−/−^* LID mice
	Picconi et al. (2003)	Abnormally high levels of phospho[Thr34]-DARPP-32	*Ex vivo*, intracellular recordings with electrodes, HFS, LFS	Unilateral 6-OHDA-induced lesion, LID rats
GPi/SNr	Prescott et al. (2014)	NO	*In vivo*, DBS	Dyskinetic PD patients
Cortex	Huang et al. (2011)	NO	*In vivo*, TBS	Dyskinetic PD patients

*All eight studies shown here reported depotentiation loss. d-Str, dorsal striatum; DBS, deep brain stimulation; HFS, high-frequency stimulation; LFS, low-frequency stimulation; cAMP, cyclic adenosine monophosphate; PKA, protein kinase A; P-DARPP-32, phosphorylated dopamine- and cAMP-regulated phosphoprotein 32 kDa; ERK, extracellular signal-regulated kinases; mTORC, target of rapamycin complex 1; RGS4, regulator of G-protein signaling 4; M4R, muscarinic M4 receptor subtype; 6-OHDA, 6-hydroxydopamine; N/OFQ, nociceptin/orphanin FQ; D-Asp, d-aspartate; TBS, theta burst stimulation; Ddo^−/−^, mutant lacking D-aspartate oxidase.

## Chronic L-Dopa-Induced Corticostriatal-Synapse Adaptations in LID

In a DA-depleted striatum that is being supplemented with L-DOPA, DA striatal levels can be preserved through sprouting of DA terminals and decreased DA uptake by DA transporters (Lee et al., 2008). Distinct degrees of DA denervation affect the induction and maintenance of two distinct forms of corticostriatal synaptic plasticity differently. Initially, DA depletion affects NMDAR-dependent LTP exclusively; with further depletion, sufficient to produce clinical symptoms, LTD is also influenced (Paillé et al., 2010).

**Figure 1:**
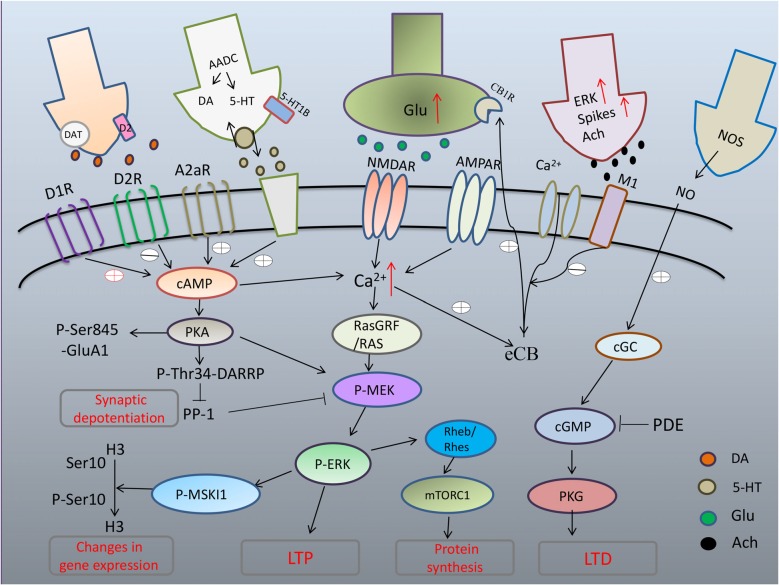
**Maladaptive corticostriatal synaptic plasticity mechanism in L-DOPA-induced dyskinesia (LID).** Excess of dopamine (DA) abnormally stimulates D1 pathway with hyperphosphorylation of extracellular signal-regulated kinase (ERK) and uncontrolled activation of protein kinase A (PKA) that leads to hyperphosphorylation of DARPP32, which blocks PP-1 causing loss of synaptic depotentiation. PKA/DARPP-32 and ERK/mitogen- and stress-activated kinase 1 (MSK1) signaling lead to phosphorylation of histone H3 in the nucleus, inducing changes in gene expression. Hyperactivation of ERK through convergent altered signaling pathways brings to increased inhibition of tuberous sclerosis complex (TSC)1/2, and consequent disinhibition of Rheb/Rhes, leading to excessive increase of signaling of mTORC1 that, in turn, exerts its long term effects through changes in protein synthesis. After chronic L-DOPA, cholinergic interneurons show increased phospho-ERK immunoreactivity and higher firing rates with increased release of acetylcholine (Ach). Striatal cGMP signaling is declined and activity-dependent LTD, which strictly relies on the nitric-oxide- (NO-) dependent activation of protein kinase G (PKG) is absent in LID.

As PD degeneration advances, nigrostriatal neurons lose their DA storage capacity, resulting in unregulated DA release and large fluctuations in extracellular DA levels (Rylander et al., 2010). The consequent large fluctuations in extracellular DA concentrations contribute to the establishment of further morphological and functional changes at both pre- and postsynaptic levels. Considerable attention has been devoted to the participation of persistent sensitization of canonical signaling downstream of D1R in the development and manifestation of dyskinesia. In striatal neurons, D_1_R activates adenylyl-cyclase through G proteins (Gαolf). Increased Gαolf levels has been associated with LID both unilateral lesion mice model (Alcacer et al., 2012) and PD patients who had received a chronic L-dopa treatment (Corvol et al., 2004). Another signaling component leading to the abnormal D1R-mediated transmission involved in LID is the adenylyl cyclase type 5 (AC5), which is highly expressed in striatal MSNs (Glatt and Snyder, 1993; Mons and Cooper, 1994). Recently, Park et al. (2014) found that AC5 knock-out mice exhibits attenuated LID by inhibition of cAMP as well as ERK signaling. In addition, several lines of evidence indicate that abnormal activation of PKA-mediated phosphorylation of DARPP-32 at T34 (Picconi et al., 2003; Santini et al., 2007; Lebel et al., 2010) and PKA dependent phosphorylation of GluA1 at Ser845 (Santini et al., 2007, 2010) in dyskinesia may have profound repercussions on synaptic plasticity (Figure 1).

Newer evidence indicates that D1R do crosstalk to glutamate signaling (mainly NMDA receptors), which are strictly correlated to abnormal synaptic plasticity and motor behavior in L-DOPA-treated dyskinetic rats. ERK dependent signaling and its downstream targets, including molecules involved in the regulation of protein translation and gene transcription, was shown to be apparently hyperactivated in DA-denervated striatum already by a single administration of L-dopa and chronic L-dopa administration (Pavón et al., 2006). L-DOPA produces pronounced activation of ERK1/2 signaling through D1 class of DA receptors. Phosphorylation of ERK1/2 and mitogen- and stress-activated kinase 1 (MSK1), a downstream target of ERK1/2, was dose-dependently blocked by the D1R antagonist, SCH23390 (Westin et al., 2007). Further to these results, (Darmopil et al., 2009) revealed that genetic inactivation of dopamine D1 but not D2 receptors inhibits LID and suppress ERK phosphorylation, phospho-acetylation of Histone H3 (pAcH3; a direct substrate of MSK-1) and FosB/ΔFosB accumulation. Recent evidence indicates that MSK1 could be involved in LID. Genetic inactivation of MSK1 attenuated LID and reduced the phosphorylation of histone H3 at Ser10 in the striatum (Feyder et al., 2016). Similarly to ERK, dopamine D1 receptor-mediated activation of the mammalian target of rapamycin (mTOR) complex 1 (mTORC1) occurs in mice that developed dyskinesia (Santini et al., 2009). Remarkably, an upstream component of the mTOR pathway, the Ras homolog enriched in striatum (Rhes), is critically involved in the pathological upregulation of mTORC1 during LID (Subramaniam et al., 2011).

In control condition, concomitant activation of DA D2 receptors and blockade of A2A adenosine receptors is able to reduce striatal glutamatergic transmission via a retrograde action of endocannabinoid-dependent mechanism (Tozzi et al., 2011). Alterations in A2A receptor expression and signaling have been observed in PD patients undergoing L-DOPA therapy (Calon et al., 2004; Ramlackhansingh et al., 2011) and in experimental models of LID (Pinna et al., 2002; Blandini and Armentero, 2012). Recently, G-protein-coupled adenosine A2A, cannabinoid CB1 and dopamine D2 receptors (A2A-CB1-D2 receptor heteromers) has been unraveled. This heteromer, present in normal and DA-depleted striatum, is however lost following acute or chronic treatment with L-dopa in rats and monkeys (Bonaventura et al., 2014; Pinna et al., 2014).

Early studies founded altered acetylcholine signaling in dopamine depletion striatum resulting in a loss of feedback control of acetylcholine release (Kayadjanian et al., 1999; Ding et al., 2006). Notably, striatal cholinergic interneurons, are involved in the D2/A2A and endocannabinoid-mediated retrograde effects. Concomitant activation of D2 DA receptors and blockade of A2A receptors reduces the firing rate of these interneurons and primary motor cortex (M1) receptor antagonism blocks the D2/A2A receptor-mediated modulation of excitatory transmission in both dMSN and iMSN (Tozzi et al., 2011). In 6-hydroxydopamine lesion mice, repeated L-DOPA treatment increases basal firing rate and stronger excitatory responses to dopamine in striatal cholinergic neurons with increased phospho-ERK immunoreactivity in this neuronal population (Ding et al., 2011). Taken together, these data suggest increased dopamine sensitivity of striatal cholinergic neurons contributes to the expression of LID.

An *in vivo* electrophysiological study demonstrated distinct effects of chronic L-DOPA administration on dMSNs vs. iMSNs in DA-depleted rats. The dMSNs had abnormally persistent cortically-evoked LTD, whereas the iMSNs exhibited LTP, rather than LTD, in response to the same stimulation (Belujon et al., 2010). Such findings suggest that LID might be caused by cell type-specific altered induction of plasticity in striatal MSNs (Calabresi et al., 2015). In a dyskinetic state, the direct pathway exhibits only LTP, while the indirect pathway exhibits only LTD. By contrast, in a parkinsonian state, the indirect pathway exhibits only LTP and the direct pathway exhibits only LTD. These pathophysiological changes are associated with a loss of bidirectional plasticity, such that only unidirectional changes in synaptic strength can occur (Thiele et al., 2014).

Apart from the major direct vs. indirect divisions of the striatal MSN population, the striatal circuitry can also be divided biochemically into two striatal compartments known as the striosomes (also known as striatal patches) and matrix. It has been proposed that neurodegenerative dysfunctions of the BG, such as LID, could involve a striosome-matrix imbalance (Crittenden and Graybiel, 2011). A recent article shows that electrically evoked dopamine release differs between the striosome and matrix compartments in a regionally-distinct manner. In the VS (ventral lateral striatum and nucleus accumbens), dopamine release in striosomes is greater than in the proximal matrix region, and in the DS (medial and lateral), the opposite is true (Salinas et al., 2016). A recent study reveals that neuromodulator substance P acting through neurokinin-1 receptor (NK1Rs) can boost DA release within the centers of striosomes, but diminish DA release in a border region where striosomes and matrix interface (Brimblecombe and Cragg, 2015). If this notion were to be confirmed with further studies, it could have explanatory implications for a range of motor and nonmotor symptoms associated with BG neurodegeneration.

## Structural Plasticity of Striatal Dendritic Spines in LID

The spiny dendrites of MSNs—the smallest processing units of biochemical signals generated at corticostriatal synapses—integrate synaptic afferents from different origins (Chen and Sabatini, 2012; Yuste, 2013). PD patients exhibit morphological changes in their striatal dendritic spines, including spine loss (McNeill et al., 1988). Using two-photon laser scanning microscopy and BAC transgenic mice, Day et al. (2006) observed that DA depletion led to a selective loss of spines and glutamatergic synapses on iMSNs, but not dMSNs.

Accumulating evidence indicates that synaptic plasticity consequent to morphologic changes in dendritic spines may be key to resolving the mechanisms underlying both PD and dyskinesia (Fieblinger and Cenci, 2015). Zhang et al. (2013) observed that PD model rats have abnormally few synapses and corticostriatal multisynaptic boutons (contacting dendritic shafts or a shaft and a spine), whereas LID model rats show a restoration in the total number of corticostriatal synapses and high densities of mushroom spines (enlarged postsynaptic densities receiving multisynaptic excitatory input). It is unclear yet whether the newly sprouted spines in the rat model form functional synapses.

Interestingly, iMSNs appear to re-grow spines that had been lost due to loss of dopaminergic innervation (Fieblinger et al., 2014; Suarez et al., 2014). The behavior of dMSNs following DA depletion, however, is less clear. Both Fieblinger et al. (2014) and Nishijima et al. (2014) did not see changes in dMSN density following a DA-denervating lesion; rather, they observed decreases in dMSN density in response to long-term L-DOPA exposure. On the contrary, Suarez et al. (2014) suggested that dMSN spine loss occurs after DA depletion, with chronic L-DOPA having no effect. It is possible that these discrepancies could be related to methodological differences, such as differences in the models or neurotoxin injection sites employed.

In summary, MSN dendrite atrophy should be regarded as a potential therapeutic target for PD. Meanwhile, LID might be the result of an L-DOPA induced mis-rewiring of corticostriatal synapses (Zhang et al., 2013; Fieblinger et al., 2014). This hypothesis should be examined further, especially with respect to cell-type specificity and the corresponding changes in intrinsic excitability (Fieblinger et al., 2014; Surmeier et al., 2014).

## Plastic Adaptations in Other Cortico-Basal Ganglion-Thalamic Circuitry in LID

The synaptic and molecular rearrangements that occur in LID have been relatively well studied in the striatum. However, plastic adaptations occurring in other parts of the cortico-basal ganglionic circuitry have received less attention. There is a growing appreciation for activity-dependent synaptic plasticity throughout the cortico-basal ganglionic loop.

The striatum is the major input station of glutamatergic innervation arising from the cortex and the thalamus. However, plastic adaptations occurring in thalamostriatal system have been poorly explored, but are likely to play an important role (Smith et al., 2014; Tritsch and Carter, 2016). Parker et al. (2016) find that dopamine depletion selectively reduces thalamostriatal drive in dMSNs mediated by AMPA rather than NMDA receptors. Combination of *in vivo* pharmacogenetics and optogenetics, the authors reveal that inhibition of thalamostriatal inputs rescues PD motor behavior, implicating maladaptive synaptic plasticity in the thalamus as playing a key role in dopamine depletion animal.

Prescott et al. (2009) reported that extrastriatal DA modulates activity-dependent synaptic plasticity in the BG output neurons of the SNr. HFS induced LTP-like potentiation of field-evoked potential amplitudes when delivered with, but not without, L-DOPA administration. Interestingly, in a more recent study, the same research group suggested that the ability of BG output nuclei to undergo depotentiation might be selectively lost in patients who develop LID (Prescott et al., 2014). Their work suggests that depotentiation in the SNr and GPi—the output n of the BG—may suppress nonessential synaptic information while integrating and normalizing signals that are to be relayed out to the thalamo-cortical network.

Ueno et al. (2014) observed that intratelencephalic-type pyramidal neurons (which project to dMSNs) in M1 of LID model rats had enlarged spines and elevated miniature excitatory postsynaptic current amplitudes. These morphological and electrophysiological changes in intratelencephalic-type pyramidal neurons in M1 could explain on a cellular level, at least in part, the loss of depotentiation-like plasticity that occurs in PD patients with LID.

Huang et al. (2011) found that depotentiation could not be induced following HFS-induced LTP-like plasticity in the M1 of PD patients with LID. Recent findings suggest that alterations in cerebellar sensory processing function may be an important contributor to maladaptive sensorimotor plasticity in M1 (Popa et al., 2013). Kishore et al. (2014) proposed that loss of M1 plasticity may reflect a loss of co-ordination among BG, cerebellar, and cortical inputs, resulting in abnormal plasticity of motor maps within M1 and, eventually, the involuntary movements characteristic of LID.

## Concluding Remarks

L-DOPA-induced changes in the plasticity of corticostriatal synapses are key to understanding the pathophysiology of LID. LID-associated changes in the synaptic and molecular biology of the striatum have been well described, whereas our understanding of activity-dependent synaptic plasticity elsewhere in the BG-thalamo-cortical network in LID is relatively undeveloped. Notably promising research areas moving forward include cell type-specific structural plasticity of striatal MSN dendritic spines, the mis-rewiring hypothesis for LID, and homeostatic adaptations in the intrinsic excitability and synaptic connectivity of striatal MSNs.

## Author Contributions

QW read related references and wrote this manuscript. WZ gave several guidance about this manuscript.

## Conflict of Interest Statement

The authors declare that the research was conducted in the absence of any commercial or financial relationships that could be construed as a potential conflict of interest.
